# Psychometric properties of the Sindhi version of the Mood and Feelings Questionnaire (MFQ) in a sample of early adolescents living in rural Pakistan

**DOI:** 10.1371/journal.pgph.0000968

**Published:** 2022-11-17

**Authors:** Janavi Shetty, Florence Perquier, Susan C. Campisi, Yaqub Wasan, Madison Aitken, Daphne J. Korczak, Suneeta Monga, Sajid Bashir Soofi, Peter Szatmari, Zulfiqar A. Bhutta

**Affiliations:** 1 Cundill Centre for Child and Youth Depression, Centre for Addiction and Mental Health, Toronto, Ontario, Canada; 2 Dalla Lana School of Public Health, University of Toronto, Toronto, Ontario, Canada; 3 Department of Psychiatry, Hospital for Sick Children, Toronto, Ontario, Canada; 4 Centre for Global Child Health; Hospital for Sick Children, Toronto, Ontario, Canada; 5 Centre of Excellence in Women and Child Health, Aga Khan University, Karachi, Sindh, Pakistan; 6 Department of Psychiatry, Temerty Faculty of Medicine, University of Toronto, Toronto, Ontario, Canada; South China Normal University, CHINA

## Abstract

There is a need for reliable and valid screening tools that assess depressive symptoms in adolescents in Pakistan. To address this need, the present study examined the psychometric properties and factor structure of a Sindhi-translated and adapted version of the child-report Mood and Feelings Questionnaire (MFQ-C) and the Short Mood and Feelings Questionnaire (SMFQ-C) in a community sample of adolescents living in Matiari, Pakistan. Questionnaires were translated into Sindhi and administered by study psychologists to 1350 participants (52.3% female) 9.0 to 15.9 years old. Measurement structure was examined using confirmatory factor analysis. Internal consistency was estimated, and convergent and divergent validity were explored using subscales from the Strengths and Difficulties Questionnaire and the Screen for Child Anxiety Related Emotional Disorders. The unidimensional structure of the MFQ-C was found to be adequate, but a four-factor structure comprising core mood, vegetative, cognitive and agitated distress symptoms best fit the data (CFI = 0.97, TLI = 0.97, RMSEA = 0.05). The original unidimensional structure of the SMFQ-C was supported (CFI = 0.97, TLI = 0.96, RMSEA = 0.07). The MFQ-C and the SMFQ-C respectively showed excellent (α = 0.92) and good internal consistency (α = 0.87) as well as satisfactory construct validity with some differences observed across the MFQ-C subscales. The SMFQ-C and the adapted MFQ-C appear to be reliable and valid measures of depressive symptoms among early adolescents living in rural Pakistan. Both total and subscale scores can be derived from the MFQ-C to assess general and specific dimensions of depressive symptoms in this population.

## Introduction

Depression in youth is common worldwide and has been identified as one of the leading causes of disability among those 10- to 24- years old [1]. However, the burden of depression and depressive symptoms in youth living in low- and middle-income countries (LMICs) is still difficult to estimate due to a lack of available data [2]. Depression in adolescence is associated with deleterious behaviours and outcomes. In LMICs, relative to non-depressed adolescents, adolescents with depressive symptoms are more likely to engage in risky sexual behaviour, substance use, delinquent activity, self-harm, and suicidal behaviour [3]. Youth who experience depression or depressive symptoms are also at a greater risk for future affective disorders [4] and ill health during adulthood [5].

Pakistan is classified as an LMIC and is the sixth-largest country in the world, with youth between the ages of 10 to 19 years old accounting for approximately 23% of the total population [6, 7]. In a school-based study of urban youth ages 11 to 18 years old living in Rawalpindi, the estimated prevalence of depression measured by the Hospital Anxiety and Depression scale was 17.2% [8]. Similar results were obtained among urban youth 12 to 14 years old living in Hyderabad: the prevalence of depression as measured by the Children’s Depression Inventory was estimated to be 14.9% in girls and 19.9% in boys [9]. To our knowledge, the only study that has examined the prevalence of depression in youth living in rural Pakistan was conducted in a sample of unmarried 16- to 18-year-old girls living in rural Punjab [10]. The estimated prevalence of clinical depression as measured by the Structured Clinical Interview for DSM-IV Disorders was 4.4% in this sample. However, one-third of the sample had high levels of psychological distress as measured by the Self Reporting Questionnaire, with higher scores being associated with poverty, physical abuse, family stress, and adverse recent life events [10].

Despite the need for mental health care, in Pakistan, the ratio of psychiatrists per 100,000 people is 0.18, which is much lower than the world median ratio of 1.27 [11]. Further, most mental health services for youth are provided in adult psychiatric facilities, located mainly in urban areas [12]. In primary care settings, many health workers have limited time and/or insufficient training to administer diagnostic interviews to all at-risk individuals [13]. Access to free, validated, and easy to administer tools for screening depressive symptoms in youth may thus contribute to the improvement of detection, surveillance, and early intervention of youth depression in LMICs.

Most existing screening tools for youth depression originate from high-income countries (HICs), and few psychometric studies have been conducted in LMICs [14]. However, the clinical presentation of depression and depressive symptoms can vary based on culture and context. Culture may define and create specific sources of distress, which in turn impact the manifestation and self-report of psychiatric symptoms [15]. For example, compared to Western cultures, Eastern cultures have been found to stigmatize mental health problems more and show more concern over the impact that disclosure of a mental illness may have on family social and economic status [16]. In some LMICs, such as Pakistan, suicidal behaviours are considered criminal offences punishable by law, which may further impact disclosure [17, 18]. Low levels of endorsement of items can then affect the validity of the measurement tool they are part of. These considerations are especially salient when attempting to administer screening tools developed in HICs in other contexts and it is unclear whether variation in prevalence estimates across countries may represent actual differences in prevalence or differences in measurement [19]. As such, there is a need for the adaptation of existing scales to cultural specificities and the investigation of the psychometric properties of screening tools prior to their use in different cultures and contexts [20]. Several indigenous and culturally adapted scales that measure adult depression are available in Urdu, Pakistan’s national language [21]. Among scales developed specifically for youth, the Strengths and Difficulties Questionnaire (SDQ) which measures internalizing more broadly [22] and the Centre for Epidemiological Studies Depression Scale for Children have been translated to Urdu [23]. To our knowledge, there are no scales available in Sindhi, the most common language spoken in the province of Sindh [24].

The Mood and Feelings Questionnaire (MFQ) is one of the most useful tests for screening childhood depression in children and adolescents [25]. It has been used and validated in many epidemiological, clinical and non-clinical studies [26–28], and is recommended by the National Institute for Health and Clinical Excellence [29]. The instrument was developed to reflect symptoms of depression specified in the *DSM-III-R*, in addition to several items of clinical relevance related to loneliness and feeling unloved [30] but has also been validated against DSM-IV(-TR) and DSM 5 depression diagnoses [31, 32]. The validated parent- (MFQ-P; 34-items) and self-report (MFQ-C; 33-items) forms are free of use [31]. Regarding the MFQ factor structure, both single- and five-factor structures (cognitive, vegetative, core mood, suicidality, and agitated distress symptoms) have been supported in HICs [19, 28, 33]. Also available is the short 13-item version of the MFQ (SMFQ), which has also shown good psychometric properties in community and clinical samples [34–36]. Although there are benefits of parents and youth as informants when assessing depressive symptoms, it has been frequently noted that parents tend to underreport their child’s depressive symptoms on screening tools [37–39]. As such, information self-reported by the adolescents themselves is often preferred [40].

To our knowledge, only two studies have examined the psychometric properties of the MFQ-C (long and short forms) in community samples of adolescents living in LMICs. In Thailand, the full-form MFQ-C was recently validated in a school-based study of youth 12 to 18 years old. The Thai MFQ-C showed excellent internal consistency (*a =* .92), convergent validity with related measures of depression (the Childhood Depression Inventory and the SDQ-emotional symptoms subscale), and good test-retest reliability [32]. However, the factor structure of the scale was not examined. In Bangladesh, the psychometric properties of the short form (SMFQ-C) were studied in a convenience sample of 9 to 17-year-old adolescents living in rural and urban areas [41]. The unidimensional structure of the SMFQ-C translated in Bangla was supported. The brief scale showed good internal consistency (*α* = .84) and adequate convergent validity with the Spence Children’s Anxiety Scale-Parent version [41].

Both the MFQ-C and SMFQ-C show limited but promising evidence of good psychometric properties in LMICs. This study aims to investigate the psychometric properties of a culturally adapted version of the MFQ-C, translated into Sindhi, in adolescents living in the rural district of Matiari, Pakistan. Given the benefits that a shorter screening tool may provide in general populations or low-resource settings, the psychometric properties of the original SMFQ-C will also be examined.

## Method

Analyses were conducted using data from the *Nash-wo-Numa* study, a cross-sectional study of adolescents living in Matiari, Pakistan, between January 2019 and February 2020. Information relevant to the current study is presented. Further details regarding sampling strategies, procedures, study measures, and objectives are described elsewhere [42].

### Participants

The participants were youth and their mothers living in Matiari. The estimated population of Matiari is 770,040 of which approximately 48,000 are school-aged children between 9.0 and 15.9 years old [24, 42]. Matiari is primarily rural, with about 85% of the population living in rural areas [24].

The primary objective of the *Nash-wo-Numa* study was to determine the prevalence of and risk factors associated with impaired linear growth in adolescents. The age ranges of participants varied between females (9.0 to 14.9 years old) and males (10.0 to 15.9 years old) to account for variation in the onset of puberty. Several steps were taken to ensure that the sample was representative of the population of Matiari. Briefly, data regarding age, sex (assigned at birth), and the number of occupants in each household obtained from a companion study were used in the selection process. Using these data, computer-assisted random sampling was employed on 53,000 households within the Lady Health Workers catchment population of 26 health facilities in the Matiari District [42]. If households had more than one adolescent, one was randomly selected to participate.

Inclusion criteria for the principal study specified that participants must be within the age ranges of the study, and their birthmothers must be available and cognitively able to participate. Female participants could not currently be or previously have been pregnant. Lastly, participants with chronic or genetic illnesses known to impact growth (e.g., heart disease, diabetes, Down syndrome) were excluded. For the present study, participants were also excluded if they had no responses to any MFQ items. Ethics approval for the principal study was obtained from Aga Khan University, Pakistan, and The Hospital for Sick Children, Canada. Ethics approval for the present study was also granted by the Centre for Addiction and Mental Health Research Ethics Committee, Canada.

### Procedure

Written consent from legal guardians and assent from participants were obtained following standard procedures [42]. Mental health measures were administered verbally by study psychologists through structured interviews conducted at the study site. Several steps were taken to address cultural sensitivities. Firstly, participant questionnaires were administered in the presence of a parent or chaperone. Secondly, four suicidality-related questions were administered only to participants who endorsed specific items and were thus identified to be at risk of self-harm. These questions were administered in a private module without a chaperone present and safety protocols were initiated by research staff if there was an indication of immediate risk of self-harm. Participants and mothers were informed that they could refuse to answer any questions. All questionnaires were translated from English to Sindhi (the primary language spoken in the province). Translations were reviewed and discussed with study psychologists and staff familiar with both languages, and pilot tested in the community [42].

### Measures

#### Mood and Feelings Questionnaires-Child Report (MFQ-C and SMFQ-C)

The original MFQ-C is a 33-item questionnaire designed to assess depressive symptoms in children and youth ages 8 to 18 [30]. Respondents are asked to rate how they have been feeling or acting recently (e.g., *“I felt miserable or unhappy”*) on a 3-point Likert scale: 0 *“not true*,*”* 1 *“sometimes true*,*”* 2 *“true*.*”* Total scores for the MFQ typically range from 0 to 66. Due to cultural sensitivities, four suicidality items from the original MFQ-C were administered only to participants who were deemed at-risk for self-harm. Therefore, the psychometric properties of our modified version of the MFQ-C was examined considering the remaining 29-item scale, with scores ranging from 0 to 58. Moreover, one school-related item (“*I didn’t have any fun in school*”), was not administered to adolescents not attending school and was conservatively coded as “0” for those participants. The Short Mood and Feelings Questionnaire (SMFQ-C) consists of 13-items from the MFQ-C that assess affective and cognitive symptoms of depression [36], with total scores ranging from 0 to 26. Higher scores indicate more severe depressive symptoms on both long and short MFQ measures.

***The Strengths and Difficulties Questionnaire (SDQ)*** is a 25-item questionnaire which has been originally validated among children 4 to 16 years old and contains five subscales: emotional symptoms, conduct problems, hyperactivity, peer problems, and prosocial behaviour [22, 43]. Higher scores indicate greater difficulties on all scales except for the prosocial behaviour subscale. The parent SDQ translated to Urdu was previously validated in an urban, clinical sample of youth ages 4 to 16 years old in Pakistan and displayed adequate discriminant validity across all subscales [22].

***The Screen for Child Anxiety Related Emotional Disorders-child version (SCARED-C)*** was used to assess symptoms of anxiety and its expected correlation with depressive symptoms. It has been validated for use in children 8 to 18 years old and consists of 41 items relating to five distinct subscales: separation anxiety, panic/somatic symptoms, social anxiety, generalized anxiety, and school avoidance [44, 45]. Items describe first-person statements (e.g., *“I am nervous”)*. Adolescents were asked to rate items on a 3-point Likert scale: 0 “*not true or hardly ever true*”, 1 “*somewhat or sometimes true*”, 2 “*very true or often true*”. The SCARED-C has not previously been validated in Pakistan, but has shown adequate face and content validity, and good internal consistency in a community sample of rural youth living in India [46].

### Statistical analyses

All analyses were conducted in R studio (RStudio Team, 2015). The significance threshold was set at *α =* 0.05. The data were assessed for missing observations. Descriptive analyses for demographic variables and scale items were conducted, including examining correlations (using Spearman correlations for ordinal variables). The Kaiser-Meyer-Olkin (KMO) measure of sample adequacy was used to assess whether the sample was adequate for factor analysis (KMO ≥ 0.60 is acceptable) [47].

The factor structure and psychometric properties of the modified MFQ-C and the SMFQ-C were assessed using confirmatory factor analyses (CFA). Due to the ordinal nature of the MFQ scale items, the mean-and-variance adjusted weighted least squares (WLSMV) estimator was used [48]. For the modified MFQ-C, we considered a unidimensional structure (29-items) as well as a four-factor solution first described by Jeffreys et al., (2016) comprising of core mood symptoms (4 items), vegetative symptoms (6 items), agitated distress (4 items), and cognitive symptoms (12 items; 26-items total). In accordance with Jeffreys et al. (2016)’s study, three items were not included in the four-factor solution (“*I ate more than usual”*, *“I did not want to see friends”*, and *“I worried about aches and pains”*). Moreover, one item originally included in Jeffreys et al. (2016)’s solution (*“she or he wasn’t as happy as usual”*) was specific to the MFQ parent version. As this item is not included in the MFQ-C, it was not considered in the present analysis. Regarding the SMFQ-C, we considered the unidimensional solution supported by previous studies [36].

Item factor loadings below 0.32 were not interpreted and a threshold of 0.70 was used to characterize items with high factor loadings [47]. Model fit was assessed using the Comparative Fit Index (CFI ≥ 0.90 indicates adequate and ≥ 0.95 indicates good fit); Tucker- Lewis index (TLI ≥ 0.90 for adequate and ≥ 0.95 for good fit), root mean square error of approximation (RMSEA ≤ 0.08 for adequate and RMSEA ≤ 0.06 for good fit) [49]. Chi-Square/Degrees of Freedom (χ^2^ /df) was reported for each model but the χ^2^ test was not used to assess model fit due to its sensitivity to large sample sizes [50]. Subgroup analyses were conducted separately for males and females and measurement invariance analyses were conducted to determine whether or not the results from the CFAs differed based on sex. Configural (no constraints), metric (constrained loadings), and scalar (constrained loadings and thresholds) models were examined. Changes in model fit indices and Δ χ^2^ were assessed for evidence of measurement invariance [49, 51].

To evaluate internal consistency, which provides an indication of the extent to which the items of a scale measure the same construct [52]. Cronbach’s alpha was calculated for the full instrument and each identified subscale. Corrected item-total correlations and Cronbach’s alpha when removing each item (*α-i*) were also assessed. Corrected item-total correlations were considered adequate when equal or higher than 0.30 [53].

Convergent and divergent validity were examined using Spearman’s rank correlations between the MFQ-C/SMFQ-C and other scales. Depression symptoms often overlap with anxiety and emotional symptoms. All have been shown to cluster together on the larger construct of symptoms of internalising disorders, separately from symptoms of externalizing disorders [54, 55]. In accordance with this conceptualization, convergent validity was assessed using the SCARED-C and the SDQ emotional symptoms subscale scores and divergent validity was examined using scores on the SDQ conduct problems, hyperactivity and prosocial behaviour subscales. Additional analyses were also conducted to compare the results of the main analysis to the results obtained in the subgroup of participants who attended school (n = 850) and assess whether school attendance may have affected our findings.

## Results

### Sample characteristics

Only one participant was excluded from our analyses due to missing responses to all MFQ items (see Fig A in S1 File for participant flow-chart). All other participants had complete data on the MFQ. The final sample consisted of 1350 participants (52.3% female).

Participants’ demographics are presented for the whole sample and by sex in Table 1. The mean age of participants was 12.4 (*SD =* 1.7) years, 12.8 (*SD =* 1.6) years for males, and 12.0 (SD = 1.7) years for females. The majority of participants attended school and lived in rural areas. Compared to males, females were younger, were less likely to attend school but more likely to live in an urban area. Their mothers were also slightly younger and they were more likely to live in a Muslim family (all *p*≤0.01, see Table 1).

**Table 1:** Demographic and family characteristics of participants.

	Participant’s Sex			
	Male (N = 644)	Female (N = 706)	Overall (N = 1350)	
Variable	N(%) or *M*[*SD*]	N(%) or *M*[*SD*]	N(%) or *M*[*SD*]	*W or* χ^2^
Participant age, years	12.8 [1.6]	12.0 [1.7]	12.4 [1.7]	*W* = 289414, ***p*<0.001**
School Attendance				
No	160 (24.8)	340 (48.2)	500 (37.0)	χ^2^_(1)_ = 77.50, ***p*<0.001**
Yes	484 (75.2)	366 (51.8)	850 (63.0)	
Urbanicity				
Urban	31 (4.8)	84 (11.9)	115 (8.5)	χ^2^_(2)_ = 21.70, ***p* <0.001**
Rural	521 (80.9)	530 (75.1)	1051 (77.9)	
Peri Urban	92 (14.3)	92 (13.0)	184 (13.6)	
Mother’s Age, years	40.2 [5.8]	39.2 [5.8]	39.7 [5.8]	*W =* 251143, ***p*<0.001**
Mother’s Native Language				
Urdu	7 (1.1)	10 (1.4)	17 (1.3)	χ^2^_(2)_ = 0.68, *p* = 0.75
Sindhi	618 (96.0)	671 (95.0)	1289 (95.5)	
Other	19 (3.0)	25 (3.5)	44 (3.3)	
Mother’s marital status				
Married	607 (94.3)	656 (92.9)	1263 (93.6)	χ^2^_(1)_ = 1.03, *p =* 0.60
Widowed/ divorced/separated	37 (5.7)	50 (7.1)	87 (6.4)	
Mother’s Parity	6.56 [2.6]	6.35 [2.5]	6.45 [2.5]	*W =* 236012, p = 0.22
Presence of Father at Home				
Living together	604 (93.8)	657 (93.1)	1261 (93.4)	χ^2^_(2)_ = 0.32, *p =* 0.85
Living elsewhere	10 (1.6)	13 (1.8)	23 (1.7)	
Died	30 (4.7)	36 (5.1)	66 (4.9)	
Mother’s Employment				
Unskilled labour	15 (2.3)	15 (2.1)	30 (2.2)	χ^2^_(6)_ = 5.14, *p =* 0.53
Skilled labour	219 (34.0)	206 (29.2)	425 (31.5)	
Agriculture	6 (0.9)	8 (1.1)	14 (1.0)	
Domestic service	8 (1.2)	12 (1.7)	20 (1.5)	
Professional	8 (1.2)	7 (1.0)	15 (1.1)	
Stay-at-home parent	381 (59.2)	446 (63.2)	827 (61.3)	
Sales/Other	7 (1.1)	12 (1.7)	19 (1.4)	
Father’s Employment				
Unskilled labour	21 (3.3)	18 (2.5)	39 (2.9)	χ^2^_(6)_ = 0.84, *p =* 0.99
Skilled labour	467 (72.5)	509 (72.1)	976 (72.3)	
Agriculture	38 (5.9)	44 (6.2)	82 (6.1)	
Sales	26 (4.0)	30 (4.2)	56 (4.1)	
Professional	58 (9.0)	67 (9.5)	125 (9.3)	
Unemployed	7 (1.1)	7 (1.0)	14 (1.0)	
Other	27 (4.2)	31 (4.4)	58 (4.3)	
Family’s Religion				
Muslim	520 (80.7)	608 (86.1)	1128 (83.6)	χ^2^_(1)_ = 6.69, ***p =* 0.01**
Hindu	124 (19.3)	98 (13.9)	222 (16.4)	

*Note*: χ^2^ = Chi-Square statistic; *W =* Wilcoxon W statistic.

### Description of the MFQ scales

The mean total score on our modified 29-item MFQ-C scale was 5.6 (*SD* = 6.6) for the total sample and ranged from 0 to 50. Mean total scores were significantly higher in females (*M* = 5.9, *SD =* 7.0) than males (*M* = 5.2, *SD =* 6.2, *W =* 173801, *p<* 0.001). The mean total score on the SMFQ-C was 2.5 (*SD* = 3.4) for the full sample and ranged from 0 to 24 with females also scoring significantly higher (*M =* 2.7, *SD =* 3.5) than males (*M =* 2.3, *SD* = 3.2, *W* = 207147, *p<* 0.001). Total score distributions for the full sample were right-skewed (MFQ-C: skew = 2.28, kurtosis = 6.65; SMFQ-C: skew = 2.18, kurtosis = 5.84). Spearman’s correlations between items are available in Fig B in S1 File.

### Factor structure of the MFQ-C and the SMFQ-C

**Fig 1:**
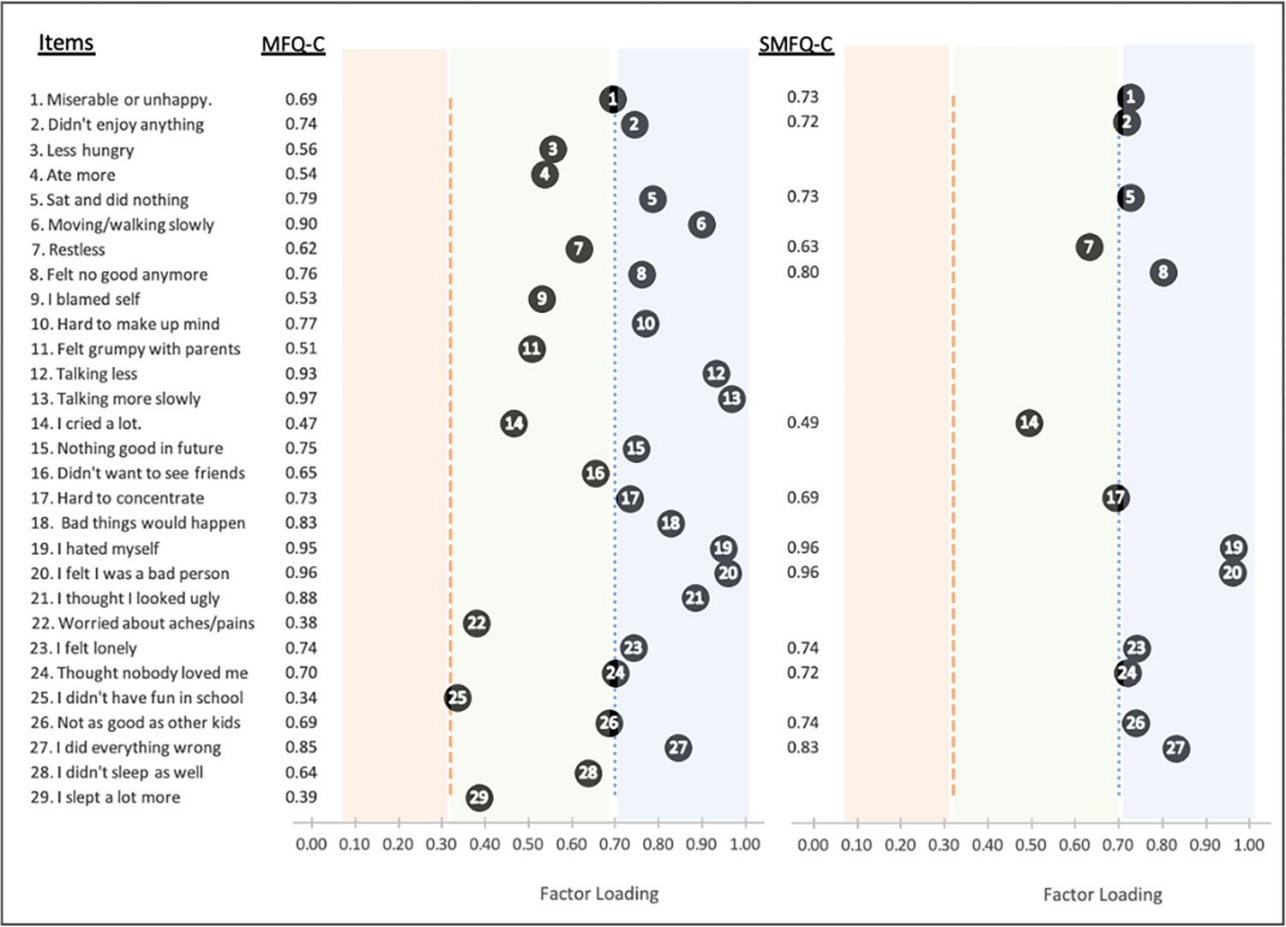
Factor loadings from the unidimensional confirmatory factor analysis of the modified Mood and Feelings Questionnaire (child-version, 29 items) and the Short Mood and Feelings Questionnaire (child-version, 13 items).

**Fig 2:**
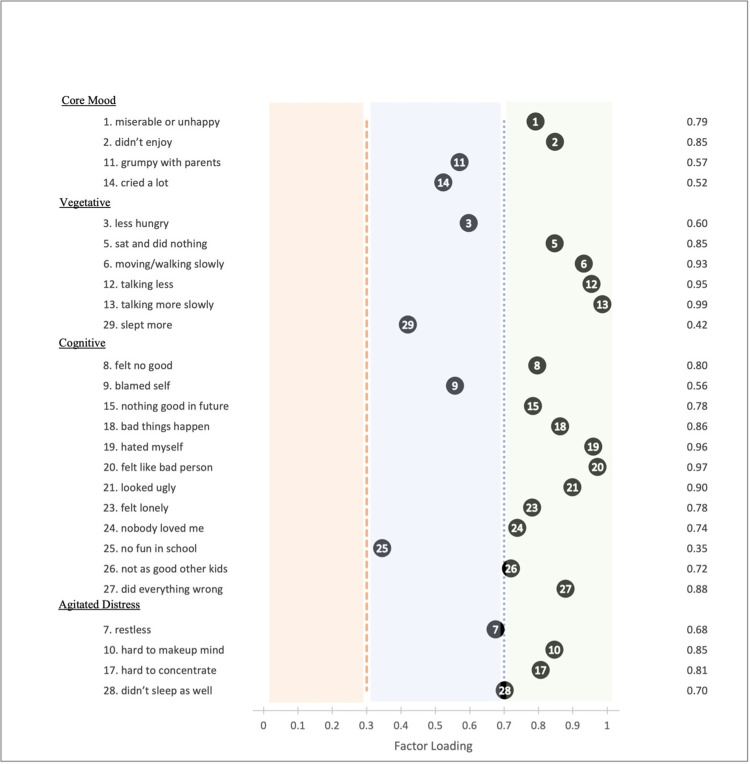
Factor loadings from the four-factor solution in confirmatory factor analysis applied to data obtained with the modified Mood and Feelings Questionnaire (child-version, 26-items).

The overall KMO measure of sampling adequacy was 0.93 for the full scale MFQ and 0.88 for the SMFQ, indicating that the sample was suitable for factor analysis. Fit indices for all models are presented in Table 2. The unidimensional structure from the original MFQ-C (29 items) showed adequate fit (CFI = 0.94, TLI = 0.94, RMSEA = 0.06) with item factor loadings ranging from 0.34 (“*I didn’t have any fun in school”)* to 0.97 (“*Talking more slowly*”) (Fig 1). However, the 26-item four-factor model showed an excellent and overall better fit than what was observed for the unidimensional factor model (CFI = 0.97, TLI = 0.97, RMSEA = 0.05). All factor loadings were greater than the specified cut-off of 0.32 [47] (Fig 2). Again, the lowest factor loading was observed for the item “*I didn’t have any fun in school*” on the ‘cognitive’ subscale (0.35). In a sensitivity analysis conducted in the subgroup of participants who attended school, this item loaded higher but still had the lowest factor loading (0.46), and there was minimal change in model fit indices. Finally, the unidimensional model for the SMFQ-C also showed excellent fit (CFI = 0.97, TLI = 0.96, RMSEA = 0.07), with all factor loadings being greater than 0.49 (Fig 1).

**Table 2:** Fit indices for confirmatory factor analyses models of the Mood and Feelings Questionnaire (MFQ-C) and Short Mood and Feelings Questionnaire (SMFQ-C).

	MFQ-C		SMFQ
Fit Index	Four-Factor (26 items)	Unidimensional (29 items)	Unidimensional
CFI	0.971	0.941	0.969
TLI	0.968	0.937	0.963
RMSEA	0.047	0.059	0.069
[95% CI]	[0.044, 0.050]	[0.057,0.062]	[0.063, 0.075]
χ^2^ /df	3.98	5.71	7.45

*Note*: Robust estimates are reported. CFI = Comparative Fit Index; TLI = Tucker-Lewis Index; RMSEA = Root Mean Square Error of Approximation; CI = Confidence Interval; χ^2^ = Chi-Square Statistic; df = degrees of freedom.

Measurement invariance across sexes was assessed for the MFQ-C four factor and for the SMFQ-C unidimensional models. There was evidence of measurement invariance. The changes in χ^2^ /DF across the configural, metric, and scalar models were non-significant and models had acceptable fit for males and females (see Table A in S1 File).

### Reliability

Cronbach’s alphas and corrected-item total correlations are presented in Table 3. Internal consistency for the total MFQ-C score was excellent (*α* = 0.92) and the corrected item-total correlations were adequate (≥ 0.30) for all items except for: *“I didn’t have any fun in school* (*r =* 0.23) and “*I slept a lot more than usual”* (*r =* 0.24).

**Table 3:** Reliability indices for items of the Mood and Feelings Questionnaire and the Short Mood and Feelings Questionnaire.

	MFQ-C		MFQ-C		SMFQ	
	(four-factor structure)		(unidimensional)*α =* 0.92		(unidimensional)*α =* 0.87	
Item	*r* ^*c*^_*it*_	*α–i*	*r* ^*c*^_*it*_	*α–i*	*r* ^*c*^_*it*_	*α–i*
**Core mood**	*α =* 0.62					
1. Miserable or unhappy	0.48	0.48	0.56	0.91	0.56	0.86
2. Didn’t enjoy	0.37	0.57	0.56	0.91	0.51	0.86
11. Grumpy with parents	0.36	0.58	0.41	0.91		
14. Cried a lot	0.38	0.57	0.35	0.91	0.36	0.87
**Vegetative**	*α =* 0.81					
3. Less hungry	0.41	0.81	0.44	0.91		
5. Sat and did nothing	0.58	0.77	0.64	0.91	0.56	0.86
6. Moving/walking slowly	0.73	0.74	0.68	0.91		
12. Talking less	0.72	0.74	0.67	0.91		
13. Talking more slowly	0.74	0.73	0.70	0.91		
29. Slept more than usual	0.24	0.85	0.24	0.91		
**Cognitive**	*α =* 0.87					
8. Felt no good	0.60	0.85	0.61	0.91	0.63	0.86
9. Blamed self	0.30	0.87	0.35	0.91		
15. Nothing good in future	0.55	0.86	0.58	0.91		
18. Bad things happen	0.60	0.85	0.57	0.91		
19. Hated myself	0.76	0.84	0.67	0.91	0.67	0.85
20. Felt like bad person	0.75	0.84	0.67	0.91	0.64	0.85
21. Looked ugly	0.66	0.85	0.61	0.91		
23. Felt lonely	0.52	0.86	0.59	0.91	0.55	0.86
24. Nobody loved me	0.50	0.86	0.49	0.91	0.50	0.86
25. No fun in school	0.17	0.88	0.23	0.91		
26. Not as good other kids	0.55	0.86	0.49	0.91	0.52	0.86
27. Did everything wrong	0.64	0.85	0.65	0.91	0.62	0.86
**Agitated distress**	*α =* 0.66					
7. Restless	0.37	0.64	0.47	0.91	0.45	0.86
10. Hard to makeup mind	0.54	0.52	0.58	0.91		
17. Hard to concentrate	0.49	0.55	0.56	0.91	0.51	0.86
28. Didn’t sleep as well	0.35	0.66	0.51	0.91		
**Items not included in the four factor solution**						
4. Ate more			0.30	0.91		
16. Did not want to see friends			0.47	0.91		
22. Worried about aches and pain			0.32	0.91		

*Note*: *r* ^*c*^_*it*_ = Corrected item-total correlation; *α =* Cronbach’s alpha*; α–i* = Cronbach’s alpha if item is removed

Regarding MFQ-C subscales, internal consistency was good for the ‘cognitive’ and the ‘vegetative’ subscales (respectively *α* = 0.87 and *α* = 0.81) and acceptable for the ‘core mood’ and ‘agitated distress’ subscales (respectively *α* = 0.62 and *α* = 0.66). Again, low item-total correlations were found for the items *“I didn’t have any fun in school”* (*r =* 0.17) on the ‘cognitive’ scale and “*I slept a lot more than usual”* on the ‘vegetative’ subscale (*r =* 0.24). Removal of these items only slightly improved the internal consistency of their corresponding subscales. In sensitivity analyses conducted among adolescents attending school, the item-total correlation for the item *“I didn’t have any fun in school*” remained modest on the unidimensional scale (*r* = 0.31) and the “vegetative” subscale (*r* = 0.28).

The internal consistency of the SMFQ-C was good (*α =* 0.87). Item-total correlations ranged from 0.36 (“*I cried a lot*”) to 0.67 (“*I hated myself*”) and Cronbach’s alpha when items were removed remain comparable (*α-i* between 0.85 and 0.87).

### Convergent and divergent validity

Convergent validity was supported by moderate-to-strong positive correlations between MFQ-C (total and subscale) scores and scores on the SDQ-emotional symptoms and on the SCARED-C subscales (Table 4). Similar results were observed for the SMFQ-C total scores.

**Table 4:** Spearman’s correlations between the Mood and Feelings Questionnaire scales and subscales and similar psychometric scales.

Scale	N	MFQ-C	SMFQ-C	Core Mood	Vegetative	Cognitive	Agitated Distress
		total	total				
**SDQ**							
Emotional symptoms	1350	0.53**	0.50**	0.46**	0.36**	0.38**	0.47**
Conduct problems	1350	0.34**	0.33**	0.39**	0.08*	0.20**	0.23**
Hyperactivity	1350	0.39**	0.36**	0.30**	0.36**	0.34**	0.34**
Prosocial behaviour	1350	-0.17**	-0.16**	-0.20**	-0.11**	-0.04	-0.10*
**SCARED**							
Panic/somatic	1336	0.57**	0.54**	0.46**	0.41**	0.45**	0.51**
Social anxiety	1323	0.40**	0.38**	0.31**	0.31**	0.31**	0.35**
Separation anxiety	1289	0.49**	0.46**	0.38**	0.37**	0.38**	0.45**
Generalized anxiety	1326	0.64**	0.63**	0.53**	0.38**	0.55**	0.55**
School avoidance	850	0.49**	0.44**	0.41**	0.37**	0.40**	0.45**

Note

* <0.05

** <0.001; MFQ-C = Mood and Feelings Questionnaire, child version (29 items); SMFQ-C = Short Mood and Feelings Questionnaire, child version (13 items); SDQ = Strengths and Difficulties Questionnaire; SCARED = Screen for Child Anxiety Related Emotional disorders.

All MFQ and SCARED scales were child-report. SDQ subscales were parent-report.

In terms of divergent validity, the MFQ-C scale, its core mood, vegetative, and agitated distress subscales and the SMFQ-C scale demonstrated a small but significant negative correlation with the SDQ-prosocial behaviour subscale (-.20 ≤ *ρ* ≤ -.10). However, no significant correlation was found between the SDQ-prosocial behaviour and the MFQ-C cognitive symptoms subscales. Surprisingly, the MFQ-C total and subscale scores, as well as the SMFQ-C scores, demonstrated low-to-moderate correlations with scores on the SDQ-hyperactivity and SDQ-conduct problems subscales (.08 ≤ *ρ* ≤ .39). The magnitude of the correlations varied greatly across the MFQ-C subscales, with a moderate correlation observed for core mood symptoms and low correlations observed for the vegetative, cognitive and agitated distress subscales.

## Discussion

Our study is the first to examine the psychometric properties and factor structure of instruments assessing depressive symptoms in early adolescents from a community sample in rural Pakistan. Though Urdu is considered the national language, Pakistan is a multilingual country with each province linked to a specific ethnic group with its own language [56]. Sindhi is the first language of over 30 million people in Pakistan [24] yet there are no psychometric scales that are available in the language which may be problematic, particularly in rural communities where bilingualism is less common. Our results showed that the modified MFQ-C and the standard SMFQ-C translated to Sindhi have good psychometric properties in a primarily rural population of adolescents.

To our knowledge, our study is also the first to examine the multiple-factor structure of the child version of the MFQ. We found the MFQ-C unidimensional structure to be adequate but the four-factor structure comprising core mood, vegetative, cognitive and agitated distress symptoms best fit the data. Past research has examined the factor structure of the MFQ-C in HICs, with all supporting the original unidimensional solution, however, alternative structures have not been considered [19, 57]. The hypothesis of a five-factor solution was developed with data collected using the MFQ-P. It was first suggested by Jeffreys et al. (2016) and has since been supported in a study based on the Spanish version of the MFQ-P, in addition to a modified 33-item single-factor structure [28, 33]. Similar to our findings, both the unidimensional and the five-factor solution fit the data well, however, a slightly better fit was observed for the five-factor solution [28].

Four items of the original MFQ-C, assessing suicidal behaviours, were not included in the modified version of the tool used in the *Nash-wo-Numa* Study. The exclusion of these items reflects the cultural norm of youth in younger age groups being accompanied by a parent or chaperone when sensitive questionnaires are administered in Pakistan or in other countries where suicidal behaviour is criminalized. Administering these questions in community samples may result in unnecessary distress and may impact accurate disclosure [17]. However, the need for this adaptation is not limited to cultural considerations and can also be necessary when adequate follow-up cannot be provided to participants who endorse suicidal items. In a study conducted in a sample of youth from the United States, three suicidality related-items of the MFQ-C were eliminated for this reason [19]. Similar to our results, the unidimensional solution was still supported for the 30-item scale [19]. Though the exclusion of suicidality items can be justified in various contexts and cultures, their exclusion prevents cross-cultural comparisons from being made when total scores are computed as normative cut-off values cannot be applied. In this case, a four-factor solution is particularly useful when examining the MFQ factor structure as it allows for the exclusion of the four suicidality items contained on a single subscale, but does not prevent reasonable comparisons to be made across the remaining subscales [33]. As such, we recommend that future users of the scale examine the subscale scores as opposed to total scores from the 29-item MFQ.

Factor loadings and item-total correlations for the item *“I didn’t have fun in school”* in both the unidimensional and four-factor structures of our modified MFQ-C were lower than those observed in HICs [28, 33]. Similar findings were noted in the subgroup of participants attending school. For comparability, we retained the item in our analyses but future research regarding the usefulness of inquiring about school-related symptoms when assessing depression in LMICs is warranted.

The reliability of the full-scale MFQ-C in this sample was similar to previously reported estimates from other LMICs (*α* = 0.90–0.92) [27, 32]. Our results were also comparable to that of studies examining the MFQ-P, with the exception of the ‘core mood’ subscale in which the internal consistency (*α =* 0.62) was lower than previously observed (*α =* 0.78) [33]. This discrepancy may be attributed to the number of items included in the ‘core mood’ subscale, which differs between the MFQ-P (five items) and the MFQ-C (four items).

Our results provide strong support for the unidimensional structure of the SMFQ-C and its reliability and validity in a sample of youth in Pakistan. The internal consistency and unidimensional factor structure of the SMFQ-C was in accordance with results obtained in past studies conducted in LMICs [41, 58]. These findings provide strong support for the use of this brief measure in community-based and/or low-resource settings in which long-form questionnaires are not feasible to administer. However, the mean SMFQ-C scores were lower in our sample compared to what was observed in a similar sample of adolescents in Bangladesh (*M =* 8.67, *SD =* 5.61) [41]. This discrepancy may reflect a true difference in the severity of depressive symptoms in adolescents living in rural Pakistan compared to those living in Bangladesh. Alternatively, the variation could be attributed to the younger age range and the use of random sampling in the present study, or because participants were not interviewed alone. Both depressive symptomatology and risky behaviours have been negatively associated with disclosure when an adult is present, thus adolescents may have underreported depressive symptoms in our study [59].

The convergent validity of our modified MFQ-C (full scale and subscales) and the SMFQ-C was supported. The observed positive correlations with scores on the SDQ-emotional symptoms subscale are consistent with past research examining the MFQ-C and MFQ-P [27, 28]. Similarly, correlations between the SCARED-C subscales and the SMFQ-C and MFQ-C total score and subscale scores were consistent with past research examining the MFQ-P [33] and are in agreement with the body of literature emphasizing the high comorbidity between depression and anxiety [60]. The SDQ-emotional symptoms and SCARED-C do not measure the exact same constructs as the MFQ-C and SMFQ-C, which explains the moderate-to-strong correlations observed. However, these correlations reflect the expected associations between depressive symptoms emotional problems and anxiety symptoms, suggesting that the MFQ-C total and subscale scores and SMFQ-C scores effectively capture the characteristics shared between these constructs.

Regarding divergent validity, surprisingly significant correlations were found between scores on the MFQ-C (full scale and subscales), the SMFQ-C, and scores on the SDQ-conduct problems and SDQ-hyperactivity subscales. However, the associations observed between depressive symptoms and conduct problems are in agreement with previous results obtained in community samples from HICs and could be explained by the overlap of symptoms of both conditions, by their mutual influence on one another, or by shared underlying etiological factors such as socioeconomic factors [61]. In LMICs, this association may be even stronger as lower socioeconomic status has been associated with both higher levels of depression [62] and conduct problems [63]. The significant correlations observed between hyperactivity and depressive symptoms has also been noted in past research [27]. This association could be attributed to the similarity in items between the two scales. The SDQ-hyperactivity subscale enquires about problems such as restlessness, fidgeting, being easily distracted, and finishing tasks [43] much of which is reflected in the MFQ-C. However, divergent validity was supported through low negative correlations between all MFQ-C scales and SDQ- prosocial behaviour, analogously to results of past research examining the MFQ-P [28].

The main strength of our work lies in the use of a representative sample and of standardized methods that limit selection and information biases and allow us to generalize our results to the population of adolescents living in Matiari. Nevertheless, our findings should be considered in light of several limitations. Firstly, because our modified version of the MFQ-C excluded four suicidal items, the total scores were computed by summing the score of 29 items, rather than 33. As such the mean total MFQ-C score obtained in our sample cannot be compared to scores obtained in other studies. Secondly, in accordance with Jeffreys et al. (2016)’s analysis, our four-factor solution did not include the following three items: “*I ate more than usual”*, *“I worried about aches and pains”* and *“I did not want to see friends”*. Modest item-total correlations were found for “*I ate more than usual”* and *“I worried about aches and pains”* when assessing the unidimensional structure of our MFQ-C (0.30 and 0.32, respectively), with similar findings also noted in a Danish school-based study [33, 38]. Further, in a study conducted in Thailand, the item-total correlation for the item “*I ate more than usual”* was also low (*r* = 0.20) [32]. This item may not be as relevant in low socioeconomic status populations as it is in HICs when assessing depressive symptoms, particularly when the prevalence of food insecurity is high. In the rural districts of the province of Sindh, food insecurity is estimated to affect 52.4% of adolescents [64]. However, we cannot exclude the possibility of these three items being clinically relevant in this population. Thirdly, due to the cross-sectional design of the study, we were unable to assess the test-retest reliability of the MFQ-C and SMFQ-C. Fourthly, since a mostly rural community sample was examined, it is unclear if these findings are generalizable to clinical or urban populations in Pakistan. Lastly, as no formal diagnostic interviews were conducted, the discriminant validity of the modified Sindhi MFQ-C and SMFQ-C could not be analyzed and meaningful cut-off scores could not be determined in our sample. Consequently, we recommend future users of these scales to consider continuous scores, in addition to dichotomized measures of depression.

## Conclusion

Overall, both the Sindhi MFQ-C and SMFQ-C appear to be reliable measures of depressive symptoms in early adolescents living in rural Pakistan. While the unidimensional structures of the MFQ-C and SMFQ-C provide a global measure of depressive symptom severity, the symptom-specific subscale scores of the MFQ-C allow for the assessment of specific dimensions of depressive symptoms. The brevity of the SMFQ-C further emphasizes its usefulness in low-resource settings, where limited time and/or trained staff prevent long-form questionnaires from being administered. Our findings also support the use of these instruments in future population-based studies, which may contribute to the surveillance and the identification of covariates of adolescent depressive symptoms in rural Pakistan. Research regarding reasonable cut-off scores that indicate probable depression in this population would provide further insight into its usefulness as a diagnostic or screening tool. More generally, the MFQ questionnaires may also be used in other LMIC populations that share similar characteristics and cultures.

## Supporting information

S1 File Fig A. Participant Flow-chart. Fig B. Spearman’s Correlations between items from the Mood and Feelings Questionnaire—child version. Table A. Measurement Invariance Analysis by Sex of the Child Report Version of the 26-item Four Factor Mood and Feelings Questionnaire (MFQ-C) and the Short Mood and Feelings Questionnaire (SMFQ-C)—child versions. Table B. Factor loadings for the four-factor and unidimensional structure of the adapted Mood and Feelings Questionnaire- child version and the unidimensional structure of the Short Mood and Feelings Questionnaire-child version.
(DOCX)

S2 File Mood and Feelings Questionnaire (Sindhi). (PDF)

S3 File PLOS questionnaire on inclusivity in global research. (DOCX)
