# Supplementary material for: Psychometric properties of the Sindhi version of the Mood and Feelings Questionnaire (MFQ) in a sample of early adolescents living in rural Pakistan
Source: PLOS Glob Public Health. 2022 Nov 17;2(11):e0000968. doi: 10.1371/journal.pgph.0000968 (PMC10021798; doi:10.1371/journal.pgph.0000968)
Supplement: S1 File — Fig A. Participant Flow-chart. Fig B. Spearman’s Correlations between items from the Mood and Feelings Questionnaire—child version. Table A. Measurement Invariance Analysis by Sex of the Child Report Version of the 26-item Four Factor Mood and Feelings Questionnaire (MFQ-C) and the Short Mood and Feelings Questionnaire (SMFQ-C)—child versions. Table B. Factor loadings for the four-factor and unidimensional structure of the adapted Mood and Feelings Questionnaire- child version and the unidimensional structure of the Short Mood and Feelings Questionnaire-child version. (DOCX) [file pgph.0000968.s001.docx]

Fig A


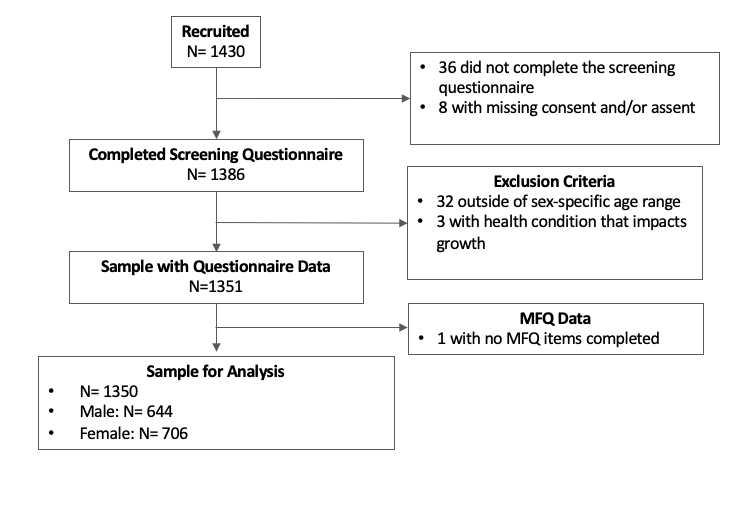


**Fig A.** *Participant Flow-chart*

Fig B


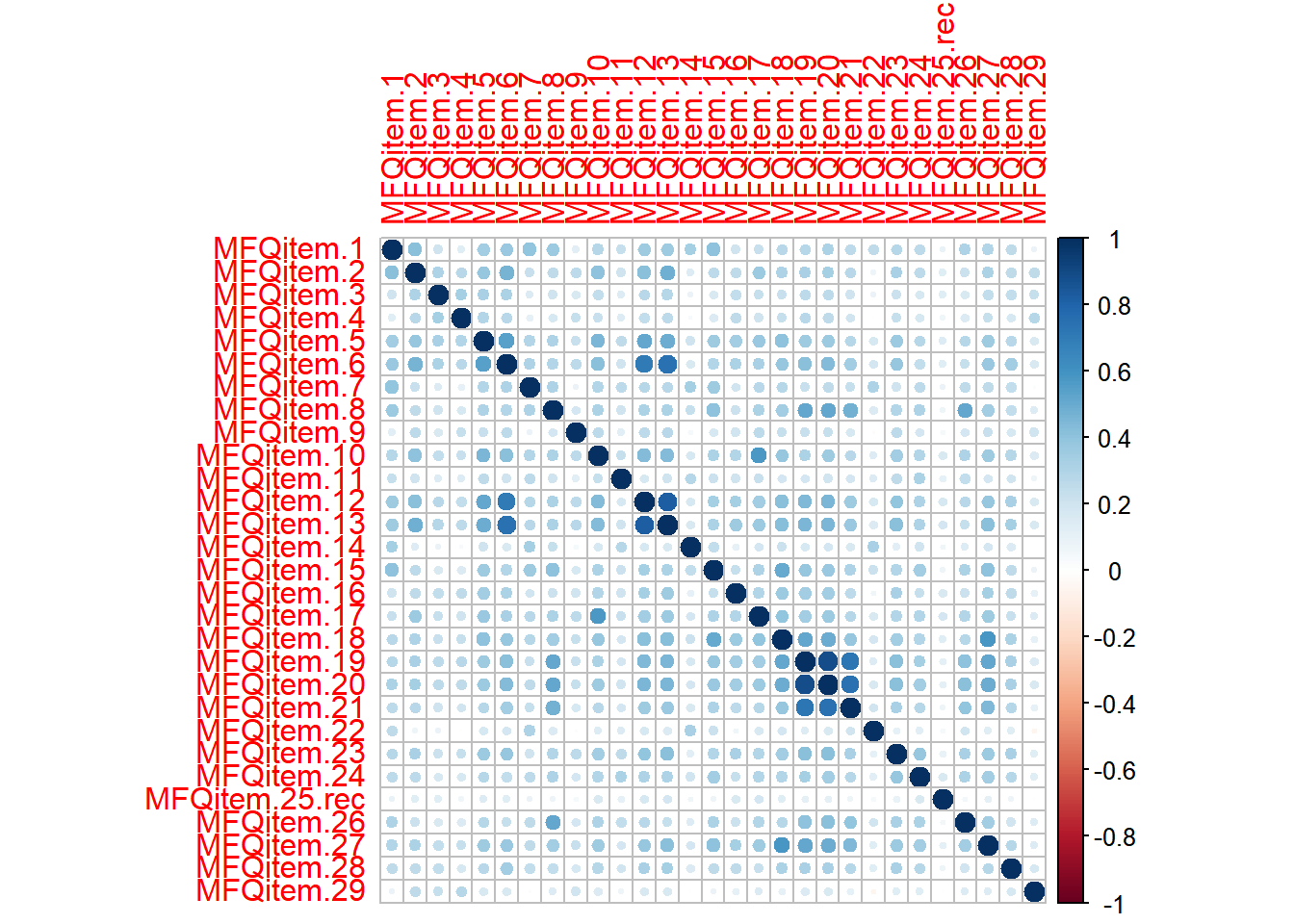


**Fig B.** Spearman’s Correlations between items from the Mood and Feelings Questionnaire - child version*.*

*Positive correlations are indicated in blue. All items were positively and significantly correlated with one another (ρ= 0.05 to 0.88) except the following: Item 29 (“I slept a lot more than usual”) was not significantly correlated with items 7 (“I was very restless”), 14 (“I cried a lot”), 22 (“I worried about aches and pains”), and 25 (“I didn’t have any fun in school”). Item 22 was not significantly correlated with item 4 (“I ate more than usual”) and item 9 (“I blamed myself for things that weren't my fault”). Lastly, item 14 was not significantly correlated with item 4.*

Table A

**Table A**. *Measurement Invariance Analysis by Sex of the Child Report Version of the 26-item Four Factor Mood and Feelings Questionnaire (MFQ-C) and the Short Mood and Feelings Questionnaire (SMFQ-C) - child versions.*

| Model | χ^2^ | df | CFI | TLI | RMSEA | Δ χ^2^ | *p-*value |
| --- | --- | --- | --- | --- | --- | --- | --- |
| MFQ-C |  |  |  |  |  |  |  |
| Configural | 1395.948 | 586 | 0.975 | 0.972 | 0.045 |  |  |
| Metric | 1228.746 | 608 | 0.981 | 0.979 | 0.039 | 19.6582 | 0.6044 |
| Scalar | 1365.547 | 630 | 0.977 | 0.976 | 0.042 | 0.7364 | 1.0000 |
| SMFQ-C |  |  |  |  |  |  |  |
| Configural | 533.95 | 130 | 0.970 | 0.964 | 0.068 |  |  |
| Metric | 430.339 | 142 | 0.979 | 0.977 | 0.055 | 6.4162 | 0.89366 |
| Scalar | 514.255 | 154 | 0.974 | 0.973 | 0.059 | 19.7541 | 0.07188 |

*Note:* Configural model includes no equality constraints, metric model sets factor loadings between groups as equal, and scalar model sets factor loadings and thresholds as equal. Delta parameterization was used. Robust estimates are reported. χ^2^ =Chi-Square Statistic; df= degrees of freedom; CFI= Comparative Fit Index; TLI= Tucker-Lewis Index; RMSEA= Root Mean Square Error of Approximation; CI= Confidence Interval.

Table B

**Table B**. *Factor loadings for the four-factor and unidimensional structure of the adapted Mood and Feelings Questionnaire- child version and the unidimensional structure of the Short Mood and Feelings Questionnaire-child version.*

| Item | MFQ-C  (four-factor structure) | MFQ-C  (unidimensional) | SMFQ  (unidimensional) |
| --- | --- | --- | --- |
| **Core mood** |  |  |  |
| 1. Miserable or unhappy | 0.791 | 0.694 | 0.727 |
| 2. Didn’t enjoy | 0.848 | 0.743 | 0.718 |
| 11. Grumpy with parents | 0.570 | 0.506 |  |
| 14. Cried a lot | 0.523 | 0.466 | 0.494 |
|  |  |  |  |
| **Vegetative** |  |  |  |
| 3. Less hungry | 0.597 | 0.556 |  |
| 5. Sat and did nothing | 0.847 | 0.785 | 0.726 |
| 6. Moving/walking slowly | 0.932 | 0.898 |  |
| 12. Talking less | 0.954 | 0.932 |  |
| 13. Talking more slowly | 0.986 | 0.968 |  |
| 29. Slept more than usual | 0.420 | 0.385 |  |
|  |  |  |  |
| **Cognitive** |  |  |  |
| 8. Felt no good | 0.796 | 0.760 | 0.802 |
| 9. Blamed self | 0.557 | 0.531 |  |
| 15. Nothing good in future | 0.784 | 0.747 |  |
| 18. Bad things happen | 0.863 | 0.827 |  |
| 19. Hated myself | 0.959 | 0.948 | 0.963 |
| 20. Felt like bad person | 0.972 | 0.960 | 0.960 |
| 21. Looked ugly | 0.899 | 0.883 |  |
| 23. Felt lonely | 0.781 | 0.741 | 0.740 |
| 24. Nobody loved me | 0.738 | 0.700 | 0.719 |
| 25. No fun in school | 0.345 | 0.336 |  |
| 26. Not as good other kids | 0.720 | 0.685 | 0.738 |
| 27. Did everything wrong | 0.879 | 0.845 | 0.830 |
|  |  |  |  |
| **Agitated distress** |  |  |  |
| 7. Restless | 0.675 | 0.615 | 0.631 |
| 10. Hard to makeup mind | 0.846 | 0.769 |  |
| 17. Hard to concentrate | 0.806 | 0.734 | 0.692 |
| 28. Didn’t sleep as well | 0.702 | 0.637 |  |
|  |  |  |  |
| **Items not included in the four factor solution** |  |  |  |
| 4. Ate more |  | 0.537 |  |
| 16. Did not want to see friends |  | 0.653 |  |
| 22. Worried about aches and pain |  | 0.380 |  |
