# Supplementary material for: Psychometric properties of the Sindhi version of the Mood and Feelings Questionnaire (MFQ) in a sample of early adolescents living in rural Pakistan
Source: PLOS Glob Public Health. 2022 Nov 17;2(11):e0000968. doi: 10.1371/journal.pgph.0000968 (PMC10021798; doi:10.1371/journal.pgph.0000968)
Supplement: S2 File — (PDF) [file pgph.0000968.s002.pdf]

MOOD AND FEELINGS QUESTIONNAIRE (MFQ)

مزاج ۽ محسوسات جي متعلق سوالات

This module is about how you might have been feeling or acting recently.

هي فارم توهان جي محسوسات ۽ ترت عمل جي باري ۾ آهي

If a sentence was not true about you, check NOT TRUE.

جيڪڏهن هڪ جملوتوهان جي باري ۾ غلط آهي ته درست نه آهي تي نشان لڳايو

If a sentence was only sometimes true, check SOMETIMES.

جيڪڏهن هڪ جملوتوهان جي باري ۾ ڪجھ صحيح آهي ته ڪنهن حد تائين درست آهي تي نشان لڳايو

If a sentence was true about you most of the time, check TRUE.

جيڪڏهن هڪ جملوتوهان جي باري ۾ صحيح آهي ته ڪنهن بلڪل درست آهي تي نشان لڳايو

		Not true<br>درست ناهي	Sometimes true<br>ڪنهن حد تائين درست آهي	True<br>بلڪل به دُرست آهي	
1.	I felt miserable or unhappy.<br>مان ناخوش محسوس ڪندو / ڪندي آهيان	0	1	2	
2.	I didn't enjoy anything at all.<br>مان شين سان لُطف اندوز نه ٿي سگهيس	0	1	2	
3.	I was less hungry than usual.<br>ون کي معمول کان گهٽ بُک لڳي هُئي	0	1	2	
4.	I ate more than usual.<br>مون معمول کان وڌيڪ کاڌو	0	1	2	
5.	I felt so tired I just sat around and did nothing.<br>مان جلد ئي ٿڪجي پوندو آهيان ۽ ڪجھ ناهيان ڪري سگهندو	0	1	2	
6.	I was moving and walking more slowly than usual.<br>مان معمول کان گهٽ هليس	0	1	2	
7.	I was very restless.<br>مان تمام گهڻو بيچين هئس	0	1	2	
8.	I felt I was no good anymore.<br>مان محسوس پئي ڪيو ته مان نيڪ ناهيان	0	1	2	
9.	I blamed myself for things that weren't my fault.<br>پاڻ انهن غلطين جو به پاڻ تي ڏوه ڏنو جن ۾ منهنجو ڪو به قصور نه هُئو	0	1	2	
10.	It was hard for me to make up my mind.<br>منهنجي لاء ڪو به فيصلو وٺڻ مُشڪل آهي	0	1	2	
11.	I felt grumpy and cross with my parents.<br>مون چڙچڙو پن محسوس ڪيو ء پنهنجي ماءّ پيءّ سان وڙهيس.	0	1	2	

12.	I felt like talking less than usual.<br>مون محسوس ڪيو ته مان معمول کان گهٽ ڳالهايو	0	1	2	
13.	I was talking more slowly than usual.<br>مان معمول کان گهٽ ڳالهائي رهيو هُئس	0	1	2	
14.	I cried a lot.<br>مون تمام گهڻو رُنو	0	1	2	
15.	I thought there was nothing good for me in the future.<br>مون کي پنهنجي مُستقبل بابت ڪا به شيء مثبت ڪونه لڳي	0	1	2	
16.	I didn't want to see my friends.<br>مان پنهنجي دوستن سان ملڻ نٿو چاهيان	0	1	2	
17.	I found it hard to think properly or concentrate.<br>مون ڪنهن به ڪم تي توجه ڏيڻ ۾ ڏُکيائي پيش اچي رهي آهي	0	1	2	
18.	I thought bad things would happen to me.<br>مون سوچو ته موسان غلط ٿي رهيو آهي	0	1	2	
19.	I hated myself.<br>مون کي پاڻ کان نفرت آهي	0	1	2	
20.	I felt I was a bad person.<br>مون محسوس ڪيو ته مان سُٺو/ سُٺي انسان ناهيان	0	1	2	
21.	I thought I looked ugly.<br>مون سوچيو ته مان بدصورت آهيان	0	1	2	
22.	I worried about aches and pains.<br>مان سورن کان پريشان رهندي آهيان	0	1	2	
23.	I felt lonely.<br>مان بلڪل به اڪيلو محسوس ڪيان ٿي	0	1	2	
24.	I thought nobody really loved me.<br>مان سمجهان ٿي ته مون سان ڪير به پيار نٿو ڪري	0	1	2	
25.	I didn't have any fun in school<br>مونکي اسڪول ۾ بلڪل به مزو نه آيو	0	1	2	
26.	I thought I could never be as good as other kids.<br>مان سمجهان ٿو ته مان ايترو سُٺو ناهيان جيترا ٻيا ٻار سُٺا آهن	0	1	2	

27.	I did everything wrong.<br>مون هر شيء غلط ڪئي	0	1	2	
28.	I didn't sleep as well as I usually sleep.<br>مان اوترو سُمهي نه سگهيس جيترو مان اڳ ۾ سُمهندو هُئس	0	1	2	
29.	I slept a lot more than usual.<br>مان معمول کان به وڌيڪ سُتُس	0	1	2	

Please thank respondent. The interview is complete.

فارم ختم ڪريو ۽ شرڪت ڪندڙ جو شڪريو ادا ڪريو
